# Psychiatry is essential for now but might eventually disappear (although this is unlikely to happen any time soon)

**DOI:** 10.1177/10398562211048141

**Published:** 2021-11-27

**Authors:** Brendan D Kelly

**Affiliations:** Department of Psychiatry, Trinity College Dublin, Trinity Centre for Health Sciences, Tallaght University Hospital, Tallaght, Dublin, Ireland

**Keywords:** psychiatry, neurology, mental disorder, epilepsy, history

## Abstract

**Objective:**

To provide an overview of specific aspects of historical and possible future trajectories of psychiatry.

**Conclusions:**

Psychiatric treatments alleviate suffering, promote physical health, and are associated with increased longevity. As the biological underpinnings of mental illnesses are slowly uncovered, they generally cease to be primarily part of psychiatry (e.g. epilepsy, anti-NMDA receptor encephalitis). If this process continues, the biological basis of all symptom-based ‘mental illnesses’ might be described, and psychiatry absorbed into neurology and other disciplines. This will be a positive development if it provides better treatment for mental illness and psychiatric symptoms in other conditions, which is psychiatry’s sole concern. Psychiatry’s own survival as a distinct discipline is irrelevant if other disciplines can do the job better, possibly in collaboration. Given the tiny impact of neuroscience on psychiatry to date, the disappearance of psychiatry is unlikely to occur anytime soon, if ever. It is possible that human psychological functioning and psychiatric suffering are sufficiently complex and changeable as to defy complete, fine-grained, neuroscientific explanation. This would leave a role for psychiatry indefinitely, treating the immensely disabling, biologically unexplained clusters of symptoms that we currently call ‘mental illnesses’, increasingly in collaboration with, or absorbed within, other disciplines in medicine.

Mental illness is common, complex and costly. Depression is a leading cause of disability worldwide, affecting over 264 million people.^1^ Almost 800,000 die by suicide each year. Despite this burden, many people cannot access treatment, including up to 85% in low- and middle-income countries.^2,3^

This is a tragedy, because psychiatry has treatments that work.^4^ Antidepressants are convincingly better than placebo for adults with major depressive disorder.^5^ Psychiatric medications are no less effective than their counterparts in general medicine: an antidepressant is more effective in reducing relapse of depression (relative risk reduction: 58%) than low-dose aspirin in secondary prevention of serious cardiovascular events (19%).^6^ There is growing evidence for psychological therapies.^7^

Treating mental illness is associated with benefits for physical health and longevity, too. While men with schizophrenia die 15 years earlier, and women 12 years earlier, than the general population, lack of antipsychotic treatment is associated with *increased* mortality.^8^ People with schizophrenia who are on antipsychotic medication have half the risk of dying during 14 years of follow-up, compared to those who do not receive antipsychotics.^9^ This includes significantly lower risk of death from cardiovascular disease as well as suicide. Cumulative mortality rates during 14 years follow-up is 46% for people with schizophrenia who are not on antipsychotics, 26% for those on any antipsychotic, and 16% for those on clozapine, after controlling for age, gender, substance abuse, medical comorbidities and other medication.

## The welcome erosion of psychiatry

Psychiatry is a paradox. We have treatments that work, but often do not have a clear understanding of why. This is because the biological basis of most mental illnesses remains obscure. If their biological underpinnings were better described, might better psychiatric treatments be designed?

While this is possible, history suggests that the discipline of psychiatry would not enjoy the benefits of such advances, because once the biological basis of a mental illness is identified, it is usually no longer considered primarily part of psychiatry. It becomes part of neurology or another discipline. Psychiatry is, perhaps, neurology without the physical signs.^10^ It is the discipline of the unexplained.

Epilepsy is a good example. Prior to the 18th and 19th centuries, epilepsy was, in effect, treated primarily as a mental illness, with natural and supernatural explanations, until a neurological basis was identified, from which point it has been considered primarily a neurological disorder.^11^ The illness, which was previously (poorly) treated by asylum doctors, is now mostly (well) treated by neurologists, sometimes in collaboration with psychiatrists.^12^ Once its biological basis was understood, epilepsy became fundamentally part of neurology, not psychiatry.

This process of attrition has continued, albeit very slowly. In the early 2000s, anti-NMDA receptor encephalitis was described, with the result that certain patients with progressive psychiatric symptoms, cognitive impairment, seizures, abnormal movements and various other complaints are now diagnosed with a condition attributable to specific autoantibodies.^13^ Approximately 90% have prominent psychiatric or behavioural symptoms, making their condition difficult to differentiate from psychiatric disorders. Many would have been previously diagnosed with, and treated for, schizoaffective disorder, depression, mania or other illnesses. Now, they are effectively treated with immunotherapy and other treatments, primarily by specialists in fields other than psychiatry.

Epilepsy and anti-NMDA receptor encephalitis are just two examples of conditions migrating from being considered primarily psychiatric conditions to being treated primarily by other disciplines, often in consultation with psychiatry. There are, however, examples of conditions migrating in the other direction, as psychiatry becomes increasingly involved in managing conditions such as intellectual disability related psychiatric disorders and neuropsychiatric aspects of Parkinson’s disease, Alzheimer’s or frontotemporal dementia. In these conditions, internal medicine physicians (paediatricians, neurologists, geriatricians) can work together with psychiatrists in shared care, as physicians often lack specific training in the management of psychiatric manifestations, such as psychosis in epilepsy or the neurodegenerative disorders.

These trends are reflected in the developing field of ‘neuro-psychiatry’, where the disciplines of neuroscience and psychiatry intertwine, and in the emerging trend towards collaborative multi-disciplinary clinical forums – such as those already existing in cancer treatment units and memory clinics, that may include specialists in both geriatric medicine and geriatric psychiatry.

Perhaps, in time, the biological basis of all conditions currently considered as ‘mental illnesses’ will be described, and psychiatry will be absorbed into neurology, geriatrics and a range of other disciplines, or will work so closely with them as to dilute psychiatry’s identity beyond recognition.^14^ That will be a very positive development if it provides better treatment for people with mental illness and psychiatric symptoms of other conditions, which is the sole concern of psychiatry. Psychiatry’s own survival as a discipline is irrelevant if other disciplines do the job better.

It should be the aim of every medical discipline to make itself redundant, through preventive measures to eliminate the diseases it treats or, as might prove the case with psychiatry, finding other disciplines that do a better job. Perhaps, someday, there will be nothing left for us psychiatrists to do, except to help out the neurologists, geriatricians and internal medicine physicians from time to time. For the moment, however, psychiatry is here to stay, if only because of the lack of better alternatives for most people with the collections of symptoms we currently call ‘mental illnesses’.

## The pace of change is glacial

There is little evidence psychiatry will be redundant any time soon. More than a century passed between the appropriation of epilepsy by neurology and recognition of anti-NMDA receptor encephalitis. The effect of neuroscience on clinical psychiatry has been tiny.^15^ Psychiatric treatment of most common mental illnesses has not been significantly impacted by the neuroscience of recent decades.

This is not attributable to lack of effort. Fields such as psychiatric genetics and neuro-imaging produce oceans of research and infinities of data, but reliably fail to impact on most clinical practice. Despite the application of state-of-the-art technology to genetic data from over one million people, there is no substantial identifiable genetic difference between schizophrenia, bipolar disorder, major depressive disorder and attention‑deficit hyperactivity disorder.^16^

The literature on neuroimaging is systematically undermined by under-powered studies and lack of replication.^17^ Much of this field might be ‘neuro’, but it is not ‘science’. It is no surprise that neuroimaging has not definitively uncovered the underpinnings of most mental illnesses or impacted significantly on most clinical practice. Given the ballooning literature, one might have expected greater impact by random chance alone.

That is not to say that the attrition of psychiatry is not occurring, but rather that it is exceptionally slow. Despite this, psychiatry is continually said to be on the cusp of a neuroscientific revolution.^18^ In 1966, Aubrey Lewis commented on the propensity of psychiatrists to rejoice ‘that they live in an age of rapid and impressive advance’.^19^ There is little reason to believe this is currently the case.

Evidence suggests that, when neuroscientific advances occur, they will slice away segments of psychiatry that will not accord with current, symptom-based ‘mental illnesses’. A deterministic biological vision would predict psychiatry’s death by a thousand cuts, as unexpected groupings of patients were appropriated by other medical specialties to receive biological treatments for identified biological anomalies.

If the current pace of change was maintained, the ultimate demise of psychiatry in this fashion would take several centuries, if not thousands of years. In the end, though, psychiatry would be left with nothing to do.

This might never occur. It is possible that human psychological functioning and psychiatric suffering are sufficiently complex and changeable as to defy complete, fine-grained, neuroscientific explanation. This would leave a role for psychiatry indefinitely, continuing to treat biologically unexplained clusters of symptoms.

In addition, it is important to distinguish between neuro-pathologically defined ‘illnesses’ and conditions that are defined by symptoms or psychosocial distress. In relation to the latter, there are many non-biological aspects of ‘mental illness’ that are more integrally germane in psychiatry than in other medical disciplines, and that include factors such as contextuality, subjectivity and meta-cognition. Some of these might permanently elude detailed neuroscientific explanation.

Physicist Emerson Pugh, in the language of the times, said that if the brain was so simple that we could understand it, we would be so simple that we couldn’t.^20^ If this is true, there will always be a role for psychiatry. If it is not, and all psychiatric phenomena are explained biologically, psychiatry might be rendered redundant by biological treatments for identified biological anomalies.

Given the current rate of progress in neuroscience, I do not expect to need to re-train as a neurologist anytime soon, but I would be happy to do so, if it helped alleviate mental illness.
